# Good Nurses

**Published:** 1874-06

**Authors:** 


### GOOD NURSES.

EVERY right minded person, especially in large cities, is grieved in reflecting upon the condition of tens of thousands of women, who, while dependent on their own individual exertions for food, and shelter, and clothing, have, comparatively speaking, so few avenues of subsistence open up to them. But there is one vocation to which they are peculiarly adapted, which is appropriate, honorable, useful and beneficent, and, withal, encouragingly remunerative, which is persistently overlooked. Good nurses for the sick are always in demand, and in cities command high wages, certainly from ten to twenty dollars a week.

A good nurse does as much toward saving the life of an invalid as a good physician; the most eminent medical men often fail of cure for want of the adjunct of a good nurse; while multitudes could be cured of very serious diseases without a particle of medicine by the offices of a judicious nurse. We happen to be personally knowing to the facts, that the sweet Alice Cary might have been living to this day, and "Phebe" too—for circumstances connected with the death of Alice killed her, with a broken heart, not disease—they loved each other so well, had not the attendant, during an improvement in her condition—well nigh wonderful—allowed the fire to go out for two nights in succession, when the weather was remarkably cold. Alice, lying on the north side of the room, became most thoroughly chilled, and one following the other so soon, she was thrown into a congested condition of the brain, became unconscious, and, in a day or two, died, having been able a very short time before, perhaps forty-eight hours, to sit up an hour or two at a time.

Any young lady of suitable qualities, by attending the lectures at the Women's Medical College in New York or Philadelphia, with the aid of private tuition, might, in a single year, qualify herself as a nurse, and make more money, and do more good than half the physicians in the land.

